# Automatic System for Visual Detection of Dirt Buildup on Conveyor Belts Using Convolutional Neural Networks

**DOI:** 10.3390/s20205762

**Published:** 2020-10-12

**Authors:** André A. Santos, Filipe A. S. Rocha, Agnaldo J. da R. Reis, Frederico G. Guimarães

**Affiliations:** 1 Programa de Pós-Graduação em Instrumentação, Controle e Automação de Processos de Mineração, Universidade Federal de Ouro Preto e Instituto Tecnológico Vale, Minas Gerais 35400-000, Brazil; andre.santos1@aluno.ufop.edu.br; 2 Robotics Lab, Vale Institute of Technology (ITV), Minas Gerais 35400-000, Brazil; filipe.rocha@itv.org; 3 Department of Control Engineering and Automation, School of Mines, Federal University of Ouro Preto (UFOP), Minas Gerais 35000-400, Brazil; reis@ufop.edu.br; 4 Department of Electrical Engineering, Federal University of Minas Gerais (UFMG), Minas Gerais 31270-901, Brazil

**Keywords:** convolutional neural network, conveyor belt, machine learning

## Abstract

Conveyor belts are the most widespread means of transportation for large quantities of materials in the mining sector. Therefore, autonomous methods that can help human beings to perform the inspection of the belt conveyor system is a major concern for companies. In this context, we present in this work a novel and automatic visual detector that recognizes dirt buildup on the structures of conveyor belts, which is one of the tasks of the maintenance inspectors. This visual detector can be embedded as sensors in autonomous robots for the inspection activity. The proposed system involves training a convolutional neural network from RGB images. The use of the transfer learning technique, i.e., retraining consolidated networks for image classification with our collected images has shown very effective. Two different approaches for transfer learning have been analyzed. The best one presented an average accuracy of 0.8975 with an F-1 Score of 0.8773 for the dirt recognition. A field validation experiment served to evaluate the performance of the proposed system in a real time classification task.

## 1. Introduction

Brazil has one of the largest mineral reserves in the world, being one of the main producers and exporters of iron ore. In this context, Brazilian mining company Vale S.A. is the world’s largest producer of iron ore and pellets, essential materials for steel-making. Technologies that improve activities related to the extraction, transportation, and/or sale of ores are of great interest.

In the mining and metallurgical sector, it is observed that the Belt Conveyor (BC) is one of the most widespread means to transport large quantities of bulk materials, which reduces the number of trucks and the cost of the shuttle services. Many bulk materials conveyed on belts are somewhat sticky. Portions of the material will cling to the conveying surface of the belt and will not be discharged with the rest of the load at the unloading point. The residual material eventually falls off at various points along the belt line, accumulating and requiring cleaning to avoid failures. The carryback can lead to excessive wear, buildup on return idlers, possible damages by forcing the belt against some part of the supporting structure, and adverse effects on conveyor operation and plant efficiency. In addition, accumulation of material on the ground or clouds of dust in the air can present a health and safety hazard [1]. Other than that, BC may have operational failures, such as longitudinal tear, deviation, and surface damage on belt rubber. Regardless the cause, when a BC is out of service in a unplanned way one can expect high maintenance costs and loss of production [2]. For Carvalho in [3], 96 h per km of BC is the average downtime per event.

In this particular work, the focus will be on the rollers of a BC. A roller is a cylinder that rotates around a central axis. It is a crucial part of a BC. Rollers generally fail in two different ways: due to locking or bearing failure, and due to the locking of the roller. The iron ore is the major contaminant of bearings and is one of the most frequent causes of their breakages [3]. Yet the excess of accumulated material in the roller supporter can lock it, which can reduce the useful life of a BC as whole.

Maintenance teams walk along the BC in regular intervals to manually inspect the rollers for accumulation of materials and other problems. This kind of activity exposes the teams to various risks, such as material projection, peer fall, fall of different levels, and exposure to weather, among others [3]. According to Yang and colleagues [4], the mechanical components of a BC do not have effective monitoring due to difficulties such as high workloads, blind spots, and other problems.

In this sense, it is interesting that intelligent solutions are developed to address this problem, preventing possible accidents, technical failures, and/or unnecessary plant shutdowns. The monitoring of the BC carried by computer vision (CV) techniques can improve the efficiency and accuracy of fault detection (see, e.g., in [2]). Nevertheless, the high cost of the installation of several cameras over miles of belts is somehow prohibitive for the vast majority of companies around the world.

The ROSI project (Robotic Device for the Inspection of Conveyor Belt Rollers) seeks to solve the problems mentioned with the development of a robotic platform equipped with a manipulator arm where a set of sensors (microphone, accelerometer, laser, and camera) will be installed to perform necessary inspections. The goal is to use the mobile robotic platform to remove the operator from dangerous tasks, see Garcia et al. [5]. In the work of Ribeiro et al. [6], the challenge is to find the best route planning for belt conveyor inspection using Unmanned Aerial Vehicles (UAV), also equipped with sensors for inspection tasks.

In this context, we propose in this paper the development of a novel and automatic visual detector that recognizes the dirt buildup on BC roller structures, using Convolutional Neural Networks (CNN), to be coupled as a service to the ROSI project as one of the inspection systems. Models based on the Visual Geometry Group (VGG) network [7], Residual Network (ResNet) [8], and Densely Connected Convolutional Network (Densenet) [9] were trained in two different scenarios with the Transfer Learning (TL) technique in order to improve the system performance. The developed models work as a binary classifier: either there is or there is not dirt buildup on the captured image, nonetheless the model is able to provide a probability for the classification, which is useful information to the maintenance team. The best scenario presented an average accuracy of 0.8975 with an F-1 Score of 0.8773 for the dirt recognition. As a proof of concept, a field validation experiment served to evaluate the performance of the proposed system in a real time classification task. The main relevance of the paper is the study and application of deep learning-based visual detectors in an industrial scenario with real data. The system itself and the obtained results are fully described in the next sections.

The remaining of the paper is structured as follows. In Section 2, we present some related work to the use of Machine Learning (ML) for classification of visual problems. Our proposed methodology is the subject of Section 3. In Section 4, we present and discuss the obtained results. Finally, the project conclusion and the future works suggestions are presented in Section 5.

## 2. Related Work

A wide range of industrial applications, such as automated monitoring, control, management, and maintenance, have been developed and deployed in recent years. The authors of [10] review the current research of Internet of Things (IoT), key enabling technologies, major IoT applications in industries, and identifies research trends and challenges. They conclude that sensors and actuators are getting increasingly powerful, less expensive, and smaller, which makes their use ubiquitous, whereas the ML approaches have been shown to provide increasingly effective solutions in areas such as scheduling, maintenance management, and quality improvement [11].

Currently, CNN has gained a high reputation in image feature extraction. According to the authors of [12], CNN has achieved state-of-the-art performance in many CV tasks but still needs keen analysis in a lot of works. They addressed an extensive survey on different learning methodologies and proved that the sparse filtering learning algorithm can outperform another learning algorithms like ICA, PCA, SRBM, and SAE.

Daily class attendance is another task that benefits from the ability to extract features from CNN. Many face recognition algorithms through deep learning have achieved promising results with large numbers of samples. The authors of [13] solved this problem using data augmentation through geometric transformation, changing the brightness of the image, and applying different filter operations. By fine-tuning the VGG model, their accuracy achieved 86.3%, outperforming PCA and LBPH. With enough training samples, their accuracy achieved 98.1%. The soil texture classification based on hyperspectral data with CNN networks is the subject of the work in [14]. Six different classifiers were compared and the validation parameters of the networks were analyzed. Among them, the CNN found similar classification results. The CNN also obtained better results in the work in [15], where pretrained models were used to analyze the viability and precision of thermal images in the pothole detection. With residual CNN models the images were correctly identified with a better accuracy of 97.08%.

Some approaches to the use of Artificial Intelligence (AI) and image processing are described in [16]. A research was done on various methods and platforms for structural inspections. In the work of Bjørlykhaug and Egeland [17], a vision system was developed for automatic quality evaluation of robotic cleaning of fish processing lines. The system was based on 10 different CNN models with augmented data for processing the images. The best result achieved by the proposed CNN approach was 99.27%. They conclude CNN approach is able to learn more complex datasets, thus producing a system that is robust to blurring, variation in contrast and poor illumination.

A camera system for detecting dust on solar panels was developed by Yfantis and Fayed [18]. They proposed a classifier based on the multivariate probability distribution function of the mode of the red, the green and the blue channel of the image. The methodology also includes the marginal distribution function of the channels. In clean panels the three-dimensional vector of the mean vector of the modes has relatively low values. When the panel gets dirty, the means of the dimensional vectors increase.

In the mining area, the work environment is complex and changeable, with the dust floating. Zhang and Zhang [19] used some edge detection algorithms to identify the phenomenon of belt longitudinal rip of BC. It was possible to suppress noise having certain fault detection credibility.

An ANN to identify belt splices was the research objective Alport of et al. [20]. Automatic splicing of a high-speed mobile BC from recorded video has been achieved to a promising degree of accuracy using wavelet coefficients as inputs to an ANN. It was concluded in the work that the wavelet algorithm more accurately discriminates splices and belt characteristics, allowing the ANN output (scaling between 0 and 1) to provide a direct measure of the confidence of this particular classification.

In order to identify mechanical failing BC, Yang et al. [2] developed a CV algorithm for segmenting BC images and detecting longitudinal and belt deviations from binary images that represent potential failures, which are a serious threat to mine safety production. After binary processing, the BC image is represented by 0 s and 1 s. Thus, the BC failure characteristics are extracted according to the 1 s distribution in the binary image.

For Yang and collaborators [4], the main mechanical components of a BC suffer from the lack of effective monitoring. They proposed an infrared thermometer inspection robot program for BC. The proposal presented was the extraction and classification of characteristics after segmentation of infrared images to automatically identify the typical elements. After data extraction, an SVM classifier was used to train the samples and perform the automatic classification of the infrared image. The correct recognition rate was up to 96.70%.

In the work of Qiao et al. [21], a proposal was presented to efficiently test the failure of the longitudinal tear of a belt. They proposed an infrared image detection method using an SVM system. The relationship between the longitudinal tear and the number of pixels of the torn area detected in the image was investigated. Thus, they established a threshold table for analyzing anti-color infrared images with a resolution of 256×256 pixels.

Carvalho et al. [3] worked on developing a method for identifying defects with the use of a drone with an embedded thermal camera. The proposal is based on the identification of the regions of interest, on a morphological processing of the images, and on radiometric data. Two algorithms for the identification of the region of the rollers were tested, the Viola and Jones and the Aggregate Characteristics Channels. As a conclusion, the algorithm for the detection of rolls reached false negative rates of up to 5% and the morphological process proved to be efficient in eliminating false positives. The drone transport platform proved to be extremely efficient with estimated productivity gains of up to 93% in the inspection time.

Olivier, Maritz, and Craig [22] used the VGG structure to characterize ore size in the mill feed by means of images. This detection is important because a very large variation in the size of the feed particles requires intervention in the system. With 223 images categorized into four classes, using transfer learning techniques and data augmentation, the results achieved were in the order of 0.97 for the F1-Score metric. The authors conclude that CNN models can outperform traditional methods when it comes to extracting the feed size distribution from the images on an industrial ore conveyor.

Naixun et al. [23] went beyond the identification of specific problems in the mining field. They were able to identify open pits in a fast and accurate way using CNN. A comparison between the recognition of the CNN with an SVM model was realized and was concluded the CNN has less misclassification, identifying almost all the details.

Computer vision is also used for visual identification of specific problems on defective surfaces. Masci et al. [24] present a supervised Max-Pooling CNN approach with steel surface defect classification. A database of a real production line obtained a rating error rate of 7.0%, working directly on the intensities of the detected and segmented steel defect pixels.

Chen et al. [25] proposed to use a multidimensional CNN to detect diverse problems in photovoltaic plate surfaces. Six different types of problems have been studied with plate surface images and RGB color space images. It was shown that deep CNN models can effectively detect solar cell surface defects, achieving an accuracy of 94.30% of defect recognition.

Besides the relevant results presented in the literature, we did not find related works to BC dirt recognition in mining. We propose a service based on deep learning, to be coupled to the terrestrial robot ROSI, that is suitable for real-time inspection systems.

## 3. Materials and Methods

This section presents the procedures performed during the data collection and construction steps of the dirt buildup detector. Two data collections on real industrial environments were performed. The first one was in the Alegria Mine in the state of Minas Gerais, Brazil. The second one was in the port of Tubarão in Espírito Santo, Brazil. In the two data collections, it was necessary to have a person in charge of the BC inspection to accompany us. This part of the work was carried out with safety procedures to avoid accidents in the field. All data collection was carried out only where we could access it.

The Python programming language was used to handle ML algorithms [26] with the aid of OpenCV and the Pytorch, a library designed to enable rapid research on ML models [27].

### 3.1. Data Collect

**Figure 1:**
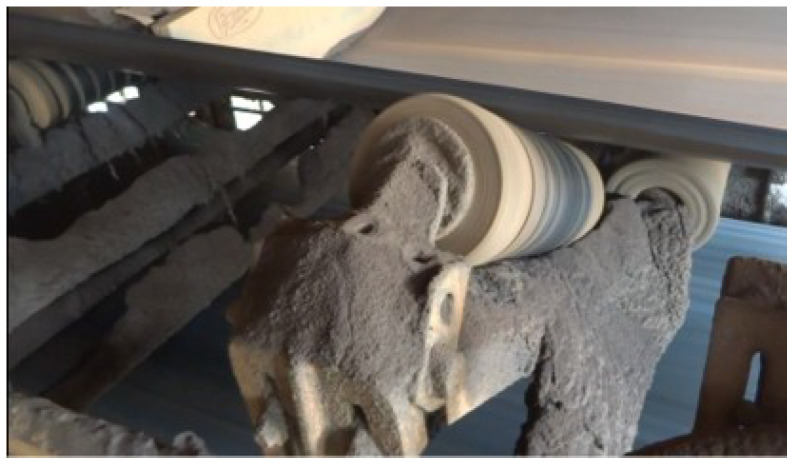
Dirt buildup on belt conveyor roller structure.

Data collection was performed to characterize and classify the problem of dirt accumulation in the structures, as shown in Figure 1.

**Figure 2:**
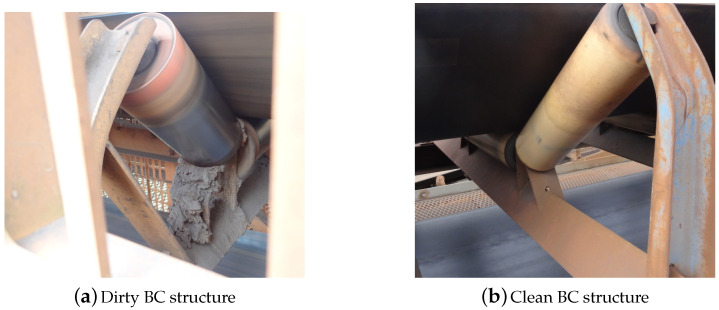
Belt conveyor roller structure (**a**) with and (**b**) without dirt buildup.

The structures of various BC were photographed with an ordinary RGB camera where the photos have dimensions of 4000×2000 pixels. Images were collected at different angles and adverse situations, such as at night and some structures with protection grids. For each photo, the roller region of the structures was extracted for it is where there is more dirt accumulation. Two selections of BC structures with and without dirt buildup are shown in Figure 2. At the end of both collections, 392 images were obtained to compose the research dataset. Thus, two main classes were defined for analysis, characterizing a binary problem as follows; (i) Clean and (ii) Dirty. With the current dataset, it is not possible to define more classes because, in a work with industrial data collection, we are limited to the characteristics of the collected data.

### 3.2. Data Preprocessing

All of the 392 images were labeled with the respective classes to compose the training and test dataset. Thus, we constructed a dataset with 228 Dirty images and 164 Clean images. Class imbalance is present in many real-world classification datasets. This issue is known to hinder the performance of classifiers which usually makes the minority class to be overlooked [28]. Thereby, the data were presented with 1.39:1 ratio in the Clean class.

**Figure 3:**
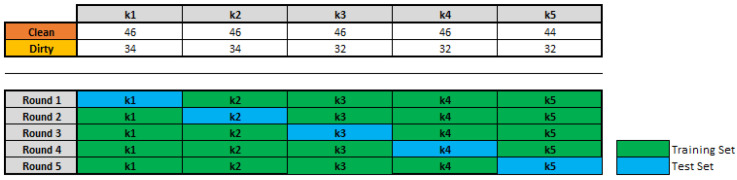
Split of the dataset to apply the k-fold cross-validation method.

For the work in [29], the k-fold cross-validation method is one of the options to have an accurate estimate of a model. We have to perform *k* rounds of learning; on each round 1/k of the data is held out as a test set and the remaining examples are used as training data. The split of the data is shown in Figure 3. We have 5 folds of data with its respective quantity of Dirty and Clean images.

One of the main challenges in ML is ensuring good performance on new unseen inputs. This ability is called generalization. To achieve that, the model capacity needs to be appropriate for the complexity of the task and the amount of training data, see in [30,31]. In practice, the amount of data we have is limited mainly because of the access security procedures on field and available time for the collection task. One way to get around this problem is to create synthetic data and add it to the training set [30]. This approach is easiest for classification and is a particularly effective technique for a specific classification problem: object recognition. This technique is called Dataset Augmentation (DA), used for instance in [15] to improve the results of their work.

We applied five types of changes into the training images as follows.

1. Random Resized Crop: applied with a probability of occurrence of 1.0, it is a random crop with the size between 65% to 100% of the original image. After that a change is applied to the aspect ratio of the cropped image between 0.75 up to 1.33. Then, the image is resized to 224 × 224 pixels to match the input size of the network.
2. Random Horizontal Flip: applied to images with a probability of occurrence of 0.5.
3. Random Vertical Flip: applied to images with a probability of occurrence of 0.5.
4. Random Rotation: applied with an angle of up to ±30° with probability of occurrence of 1.0.
5. Color Jitter: inserted a random change of up to 0.05 in hue and saturation, with a probability of occurrence of 1.0.

**Figure 4:**
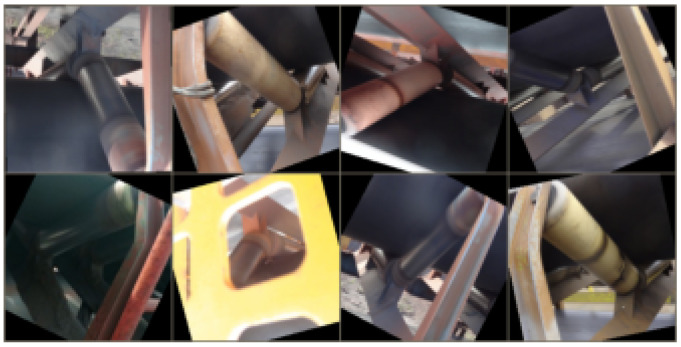
Preprocessing and data augmentation applied to images in training time.

All transformations can be implemented by pytorch and were performed during training on each data batch according to the mentioned probabilities. A batch of data with DA can be seen in Figure 4.

### 3.3. Network and Training Definition

For the training it was first necessary to define a network model for pattern recognition in images. The models chosen for the classifier were the VGG16, ResNet18, and Densenet161.

The benchmark carried out in [32] showed precision that does not improve with the complexity of the model in a linear way and not all models use their parameters with the same level of efficiency. When testing 44 different models, they evaluated Top-1 and Top-5 accuracy on the ImageNet-1k, model complexity by the total amount of learnable parameters, memory usage, computational complexity by considering the floating-point operations, and inference time. VGG16 is one of the most computationally complex architectures with a top-5 accuracy of approximately 90%. Resnet18 and Densenet161 have less complex architectures, but with an accuracy of approximately 88% and 94%, respectively. For memory consumption, the ResNet18 model was one of the least consumed memory of an NVIDIA Titan XP card for a batch of 8 images, with 0.69 GB, while the Densenet161 and VGG16 models consumed 0.80 and 1.80, respectively. Comparing different network models like these in a specific task, such as mining inspection activities, can provide an overview of how these systems can be incorporated into industrial activities.

According to the works in [7,8], the VGG was originally trained with the ImageNet dataset and presents great generalization characteristics for several problems. The VGG architecture had a significant error drop compared to the previous state of art network architectures. The model has an architecture with very small (3 × 3) convolution filters and the VGG16 has a depth of 16 layers. The authors of [8] presented the residual learning framework. They provided evidences showing that these residual networks are easier to optimize, and can gain accuracy from considerably increased depth. An ensemble of these residual nets achieves 3.57% error on the ImageNet test set and won the 1st place on the ILSVRC 2015 classification task. ResNet18 is a CNN that is 18 layers deep. The Densenet is presented in [9]. The authors connected all layers of the CNN directly with each other to guarantee maximum information flowing between layers. This made it possible to have fewer parameters than traditional CNNs. With the connections between all layers, the final classifier makes a decision based on all feature maps in the network. The comparison presented in [9] shows that Densenet networks perform better than ResNet models on top-1 error rates on the ImageNet validation dataset.

The TL technique was used in the models as a starting point for the proposed solution [33]. TL refers to the situation where what has been learned in one environment is exploited to improve generalization in another environment [30] and represents progress towards making machine learning as efficient as human learning [34].

**Figure 5:**
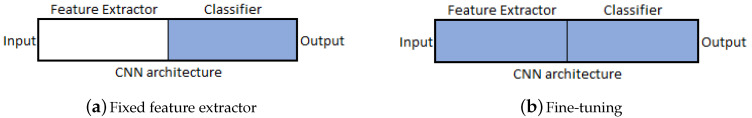
Scenarios presented for the training of the models. The blue blocks indicate the weights that were trained.

As the proposed problem has two classes, the structure of the networks has been altered to satisfy the classification conditions. The output layer of the models have been changed to have two neurons, which represent the classes of the dirt buildup classifier: Clean and Dirty. Two main scenarios of the TL technique, as described in [35], were used to compare the trained models:

1. **CNN as fixed feature extractor.** The trainable weights are frozen for all of the network except that of the fully connected (FC) layers. The last FC layer (output layer) is replaced with a new one with two neurons and random weights to match the number of classes in the problem. Only the FC layers are trained. A representation of this scenario is shown in Figure 5a, where the blue block indicates that only the classification weights are trained. In this scenario the original feature extractors of the models are used to extract the main features of the data. These features run through the FC layers trained from scratch.
2. **Fine-tuning the CNN.** Instead of random initialization, the network is initialized with the pretrained weights of the model. Only the last FC layer (output layer) is randomly initialized with two neurons to match the number of classes in the problem. During the training, all the weights of the network (convolutional and classifier layers) are retrained. A representation of this scenario is shown in Figure 5b, where the blue blocks indicate that all weights in the network are trained. In this scenario, the feature extractors of the models are trained along with the FC layers.

In [36], the TL is a common and highly effective approach to deep learning on small datasets. The author states that the levels of representations extracted depend on the depth of the layer: The first layers extract generic features, whereas the last layers extract more abstracts features from the data. Here, we compare these two techniques to have more accurate information about the dirt buildup recognition in the mining field.

The training of the models was set in 80 epochs and the k-fold cross-validation method was performed to observe each scenario of the TL. The main training parameters are presented in Table 1. The cross entropy loss function can be described as Equation (1):

(1) $$loss\left(x,class\right)=-x\left[class\right]+\log\left(\sum_{j}\exp\left(x\left[j\right]\right)\right)$$

where *x* is the input data, class is the target class, x[class] can be interpreted as the CNN score for the positive class, and x[j] is the score for all *j* classes of the model Clean and Dirty). This criterion combines the Log Softmax and the Negative Log Likelihood Loss function.

### 3.4. Field Validation

A field validation was performed as proof of concept. To this end, a new visit was made at the Tubarão port. This procedure was useful to check the system in a real time classification simulating a robot inspection.

**Figure 6:**
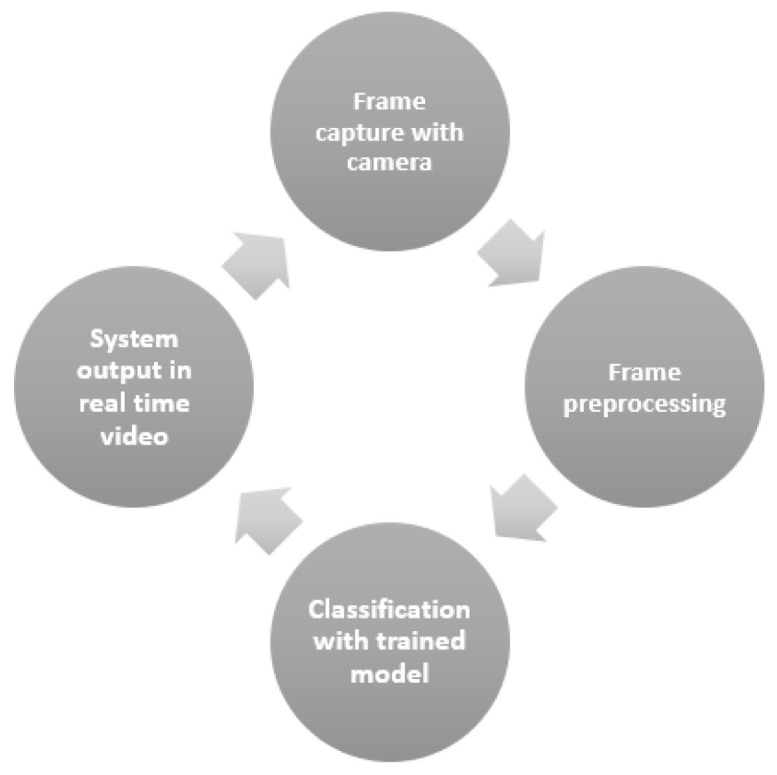
Field validation procedure loop.

The validation process was performed with a Logitech c920 Pro Full HD webcam connected to a notebook. In Figure 6, it is possible to observe the loop that represents the simplified procedure implemented.

## 4. Results and Discussion

This section will present the results of the tests performed and the system validation discussions.

### 4.1. Model Evaluation

Both TL scenarios will be analyzed along with the Precision, Recall, and F1-score metrics, see Equations (2)–(4), respectively. To do so, it is important to set the False Negatives (FN), True Positives (TP), False Positives (FP), and True Negatives (TN).

(2) $$Precision=\frac{TP}{TP+FP}$$

(3) $$Recall=\frac{TP}{TP+FN}$$

(4) $$F1=2\cdot \frac{Precision\cdot Recall}{Precision+Recall}$$

#### 4.1.1. CNN as Fixed Feature Extractor

The test results of the k-fold cross-validation method for the models can be seen in Table 2. Accuracy indicates how well the model got from the possible predictions. With the five rounds, the mean values and standard deviation of loss function and accuracy for the k-fold cross-validation were calculated as shown in Table 2.

For each model, we analyzed the main metrics presented in Equations (2)–(4). Taking the Dirty class as the positive: precision represents the proportion of images with dirt buildup who were correctly classified as belonging to Dirty. The recall represents the proportion of images with dirt buildup who were correctly classified into the samples that should be classified as with dirt buildup. The F1-score is the harmonic mean of precision and recall. The mean for all models are presented in Table 3 taking the Dirty class as positive for comparison.

**Figure 7:**
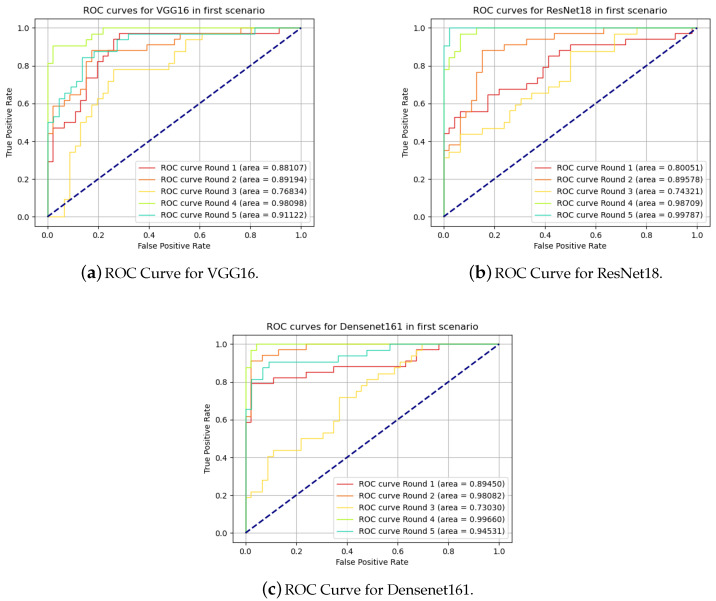
Receiver Operating Characteristic curves for in first scenario.

Another alternative to evaluate a binary prediction model is the use of the receiver operating characteristic (ROC) curve. In [37], its initial use was initially reported in comparison of algorithms and extended even in proposing new algorithms. The performance of the model can be plotted on a two-dimensional graph with the rate of true positives as a function of the rate of false positives. The classification ranking to generate the graph was performed based on the probability of the result belonging to the Dirty class. The graphs for the three models are presented in Figure 7.

#### 4.1.2. Fine-Tuning the CNN

The same approach was applied in the second scenario. The test results of the k-fold cross-validation method for the models can be seen in Table 4. All models had better results in comparison with the first scenario. The average accuracy improved by 10.2610pp for VGG16, 8.0890pp for ResNet18, and 6.1750 for Densenet161.

For the evaluation of the metrics, the results are displayed in Table 5. Taking the Dirty class as positive, and comparing with the first scenario, we have an improvement of the VGG16 and ResNet18 models by 18.1920pp and 10.56pp, respectively, for the F-1 Score. The Densenet had the better results for Recall and Precision in first scenario, thus the F1-Score improved by 6.62 in the second one. The accuracy is in agreement with the result presented in [32], where the Densenet model had the best result among the three models used in this work.

**Figure 8:**
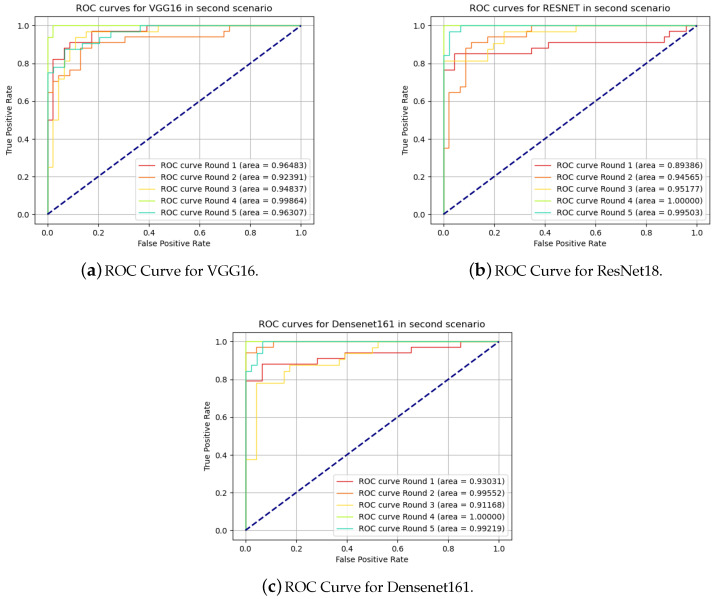
Receiver operating characteristic curves in second scenario.

The ROC curves for the second scenario are presented in Figure 8.

#### 4.1.3. Discriminative Localization

Recent works, e.g., in [38], have shown that CNN has the ability to locate objects in images without knowing their location beforehand. To learn deep features for discriminative localization, Zhou et al. [39] proposed a technique for generating Class Activation Maps (CAM) using the Global Average Pooling (GAP) on CNN. The CAM allows the visualization of the predicted class scores on any given image, highlighting the discriminative object parts detected by the CNN model. The CAM for class *c* is given by Equation (5). This is achieved by projecting back the weights of the output layer on the convolutional feature maps.

(5) $$M_{c}\left(x,y\right)=\sum_{k}w_{k}^{c}f_{k}\left(x,y\right)$$

where $w_{k}^{c}$ is the weight corresponding to class *c* for unit *k* and $f_{k}\left(x,y\right)$ represents the activation of unit *k* in the last convolutional layer at spatial location (x,y).

**Figure 9:**
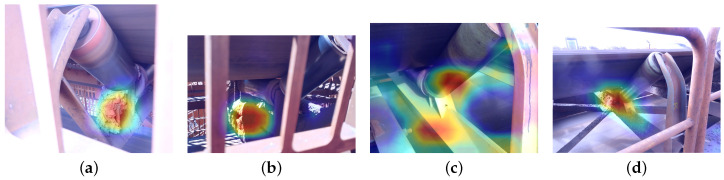
Class Activation Maps applied on the best model. Panels (**a**–**d**) show where the network is looking.

We used the CAM technique as a way of visual validation of the results. Some of the outputs for the best scenario are shown in Figure 9. We can see that the networking is looking at the area of interest. The color red indicates the area that was most activated by the network. In faint blue we have the region that has less importance on the classification.

### 4.2. Field Validation

Field validation served as a proof of concept for the system to be integrated as a service into a robotic inspection system. As the best result was achieved with the second training scenario model, a trained model in the second scenario was used in the field tests.

**Figure 10:**
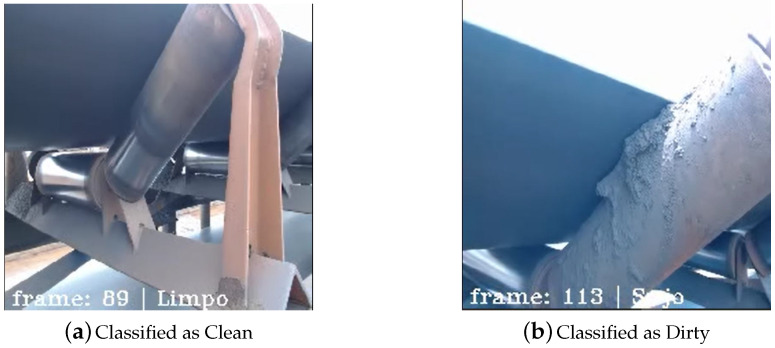
First field validation frames showing a Clean classification in (**a**) and a Dirty classification in (**b**).

At the port, 30 recordings were made for real-time classification, with 15 recordings for each class. Some of the first tests can be seen in Figure 10 that shows two frames of two different recordings.

It was interesting to note this classification because the second image classified as Dirty has a specific feature that was not present in the training data: The dirt between the upper BC roller surface and the bottom surface of the conveyor belt rubber. Most training images have dirt on the bottom structure that supports the roller. This demonstrates the generalization capability of the model.

**Figure 11:**
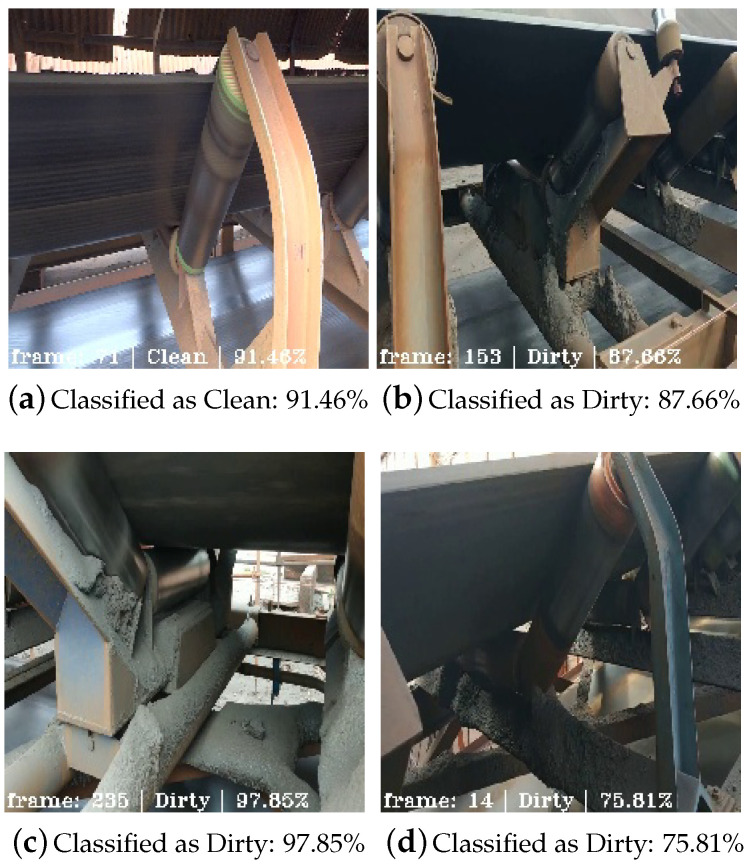
Field validation frames with real-time rating probabilities.

To have more information in the field validation, the system was implemented to show the classification probability as a system confidence indicator to the user, as shown in Figure 11.

In Figure 11a, it is possible to see a classification as Clean with a high probability of 91.46%. Figure 11b,c present classifications with high probability as well, but this time recognizing the dirt buildup on BC structures, respectively, 87.66% and 97.85%. Figure 11d has been shown to indicate a correct classification, but with lower probability, which is within the expected range given the results shown in Table 5. Even dirt on a not so crowded level can be recognized with reasonable probability. This can be an asset for the maintenance team to define when and what structure must be cleaned.

For further validation details, some videos from images presented can be accessed as supplementary material to the manuscript for viewing results and viewing how the model behaves with real-time variations.

**Figure 12:**
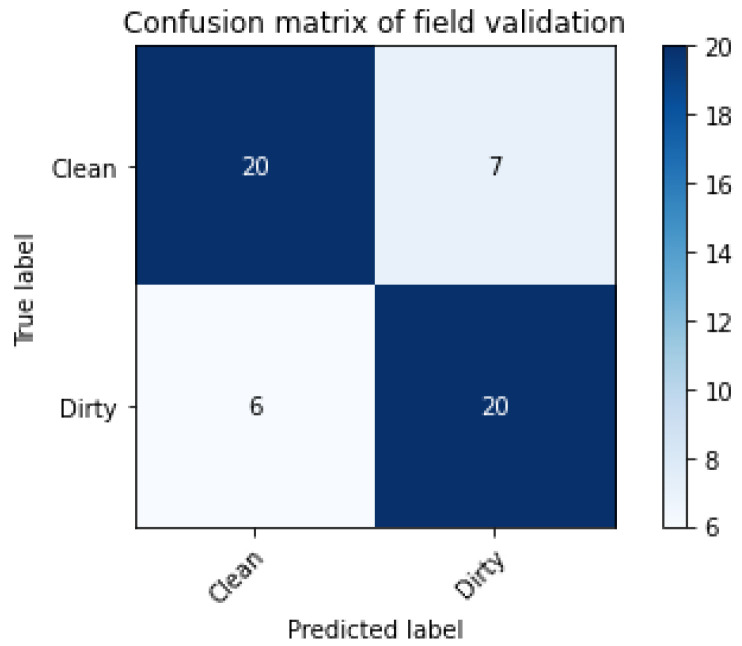
Field validation confusion matrix.

With the 30 validation videos, the rolls on which the recording was most stable, without flicker, were observed, see Figure 12. Thus, the confusion matrix was observed. For the recall, precision, and F1-Score equations, the results for the Dirty class were 0.77, 0.74, and 0.75, respectively.

At last, we took the VGG16 and Densenet161 from the second scenario, and performed 30 rounds with 100 frames of a video to analyze how much time it took to process the frames and get a classification. The average of the frames per second (FPS) calculated was 3 and 1 FPS, respectively. The experiment was realized on a Intel^®^ Core™ i5-7200U CPU @ 2.50GHz × 4 with 7.6 GiB memory and Intel^®^ HD Graphics 620.

## 5. Conclusions

Automatically detecting dirt buildup using ML can assist in the BC structures inspection process by decreasing uptime, aiding decision-making in maintenance sectors. The use of the TL technique proved to be efficient for this type of problem, enabling effective training in the recognition of the desired characteristics of dirt buildup in BC roller structures.

Taking the second scenario into account, we could see that the results were very close. This is in line with the work in [32], which concluded there is no relationship between model complexity and accuracy. Even for a specific kind of problem, like dirt buildup, we did not perceive a linear relationship. The more complex model, VGG16, had the least accuracy very close to the Resnet18, the model with least complexity, while the model with intermediate complexity, DenseNet161, had better accuracy.

The VGG16 presented better recall on all scenarios for the Dirty class but had the smaller precision. The model can be used in the field for a higher quality dirt identification. The F1-Score of densenet161 showed the model had a higher balance between precision and recall. Densenet161 can be the best solution if the identification of the clean rollers is highly requested. ResNet18 model, with the intermediate F1-Score, can be indicated for situations when the computational capacity is limited.

The training of all model weights proved to be more appropriate. As we want to recognize the dirt build-up, those improvements are important to state the effectiveness of fine tuning the model. The F-1 score improved for all classes comparing the scenarios. To achieve better results it is important to collect more data in adverse situations, such as at night and with grid protections.

With the functional inspection system, field validations were successfully performed. One can apply such solution in fixed or mobile systems, such as terrestrial robot [5] or a UAV [6].

### Future Work

To deal with inspection difficulties, the mining industry has been doing a lot of work to automate the various inspection services through robotic devices [5]. A robotic platform equipped with a manipulator arm and a set of sensors in order to perform inspections of BC is under development. Our proposed classifier will be prepared to be embedded as a service in ROSI platform in a near future.

## Figures and Tables

**Table 1:** Parameters of the training for the dirty classifier.

Parameter	Description
Batch size	8
Optimizer	Stochastic Gradient Descent (SGD)
Learning rate	0.001
Decay	0.1
Step size	65
Loss function	Cross Entropy Loss

**Table 2:** Loss and Accuracy of the k-fold cross-validation method for models in the first scenario.

Rounds	VGG16		ResNet18		Densenet161	
	Loss	Accuracy	Loss	Accuracy	Loss	Accuracy
1	0.49467	0.73750	0.55240	0.76250	0.42724	0.85000
2	0.52849	0.72500	0.49380	0.72500	0.34067	0.85000
3	0.62515	0.70513	0.62907	0.64103	0.64232	0.62821
4	0.55003	0.93590	0.30824	0.94872	0.23751	0.97436
5	0.52007	0.73684	0.30650	0.86842	0.47549	0.77632
Average	0.54368	0.76807	0.45800	0.78913	0.42465	0.83578
Standard Deviation	0.04443	0.08473	0.13026	0.10818	0.13568	0.12576

**Table 3:** Mean of the metrics for the first scenario.

Network	TP	FP	FN	TN	Precision	Recall	F-1 Score
VGG16	16.8	16	2.2	43.4	0.51220	0.88421	0.64865
Resnet18	22	10.8	5.8	39.8	0.67073	0.79137	0.72607
Densenet161	25.2	7.6	5.2	40.4	0.76829	0.82895	0.81110

**Table 4:** Loss and Accuracy of the k-fold cross-validation method for models in the second scenario.

Rounds	VGG16		ResNet18		Densenet161	
	Loss	Accuracy	Loss	Accuracy	Loss	Accuracy
1	0.32579	0.82500	0.40805	0.85000	0.37880	0.91250
2	0.56521	0.78750	0.38419	0.83750	0.17310	0.96250
3	0.34537	0.85897	0.38491	0.83333	0.48134	0.73077
4	0.08021	0.98718	0.05477	0.98718	0.08661	0.98718
5	0.35257	0.89474	0.32437	0.84211	0.23900	0.89474
Average	0.33383	0.87068	0.31126	0.87002	0.27177	0.89753
Standard Deviation	0.15389	0.06825	0.13120	0.05884	0.14175	0.08978

**Table 5:** Mean of the metrics for the second scenario.

Network	TP	FP	FN	TN	Precision	Recall	F-1 Score
VGG16	25	7.8	2.4	43.2	0.76220	0.91241	0.83057
Resnet18	25.2	7.6	2.6	43	0.76829	0.90645	0.83167
Densenet161	28.6	4.2	3.8	41.8	0.87195	0.88272	0.87730

## Supplementary Materials

The supplementary materials are available online at: https://www.mdpi.com/1424-8220/20/20/5762/s1.
